# Altered functional connectivity of insular subregions in subjective cognitive decline

**DOI:** 10.3389/fnhum.2024.1404759

**Published:** 2024-05-27

**Authors:** Huan Tian, Weimin Zheng, Junkai Wang, Shui Liu, Zhiqun Wang

**Affiliations:** ^1^Department of Radiology, Aerospace Center Hospital, Beijing, China; ^2^Department of Radiology, Beijing Chaoyang Hospital Affiliated to Capital Medical University, Beijing, China

**Keywords:** insula, network, functional connectivity, fMRI, subjective cognitive decline

## Abstract

**Objective:**

Recent research has highlighted the insula as a critical hub in human brain networks and the most susceptible region to subjective cognitive decline (SCD). However, the changes in functional connectivity of insular subregions in SCD patients remain poorly understood. The present study aims to clarify the altered functional connectivity patterns within insular subregions in individuals with SCD using resting-state functional magnetic resonance imaging (rs-fMRI).

**Methods:**

In this study, we collected rs-fMRI data from 30 patients with SCD and 28 healthy controls (HCs). By defining three subregions of the insula, we mapped whole-brain resting-state functional connectivity (RSFC). We identified several distinct RSFC patterns of the insular subregions. Specifically, for positive connectivity, three cognitive-related RSFC patterns were identified within the insula, suggesting anterior-to-posterior functional subdivisions: (1) a dorsal anterior zone of the insula that shows RSFC with the executive control network (ECN); (2) a ventral anterior zone of the insula that shows functional connectivity with the salience network (SN); and (3) a posterior zone along the insula that shows functional connectivity with the sensorimotor network (SMN).

**Results:**

Compared to the controls, patients with SCD exhibited increased positive RSFC to the sub-region of the insula, demonstrating compensatory plasticity. Furthermore, these abnormal insular subregion RSFCs are closely correlated with cognitive performance in the SCD patients.

**Conclusion:**

Our findings suggest that different insular subregions exhibit distinct patterns of RSFC with various functional networks, which are affected differently in patients with SCD.

## 1 Introduction

Subjective cognitive decline (SCD) is considered the initial clinical sign in the Alzheimer’s disease (AD) continuum (Jessen et al., 2010). Patients with SCD often express concerns about their memory, attention, and other cognitive functions, even though their performance on standardized cognitive tests may fall within the normal range (Jessen et al., 2014). SCD is characterized by a self-perceived decline in cognitive function without any objective impairment on standardized neuropsychological tests and is linked to an increased risk of progressing to mild cognitive impairment and dementia (Molinuevo et al., 2017). Currently, the diagnosis of SCD relies largely on self-reported deterioration in memory or other cognitive functions (Jessen et al., 2020). Due to the lack of objective and sensitive biomarkers, symptoms at the SCD stage are often dismissed as normal age-related changes. Pathophysiological changes in the brain have been shown to occur long before cognitive symptoms manifest (Morris, 1993). Thus, investigating biomarkers for SCD could aid in the early detection of AD. Functional connectivity refers to the temporal correlation of neuronal activity between different brain regions, indicating the coordination and integration of information processing in the brain. Altered patterns of functional connectivity have been observed in individuals with various neurodegenerative disorders. The insula is a crucial brain region involved in various cognitive and emotional processes, such as interoception, emotional awareness, and decision-making. Recent studies suggest that altered functional connectivity within the insula may contribute to the initial stages of cognitive decline and dementia. Despite the increasing interest in the insula’s role in cognitive decline, relatively few studies have specifically explored the functional connectivity of insular subregions in individuals with SCD. Understanding the functional connectivity patterns within the insula among individuals with SCD could offer valuable insights into the underlying neural mechanisms of subjective cognitive decline and help recognize potential biomarkers for early detection and intervention.

Therefore, it is necessary to explore whether other brain function changes occur in patients with SCD. Resting state functional magnetic resonance imaging, which can indirectly reflect neuron activities by measuring the blood-oxygen-level-dependent (BOLD) signals, is promising in the early detection of SCD. The insula is an important brain region of the frontolimbic system, located at a key node in the salience network. It integrates external sensory information with internal emotional and homeostatic signals to coordinate dynamic changes in the brain network. The insula is a multifaceted brain structure that can be subdivided into anterior and posterior regions, each with distinct functional properties (Kurth et al., 2010). Previous research has suggested that altered connectivity within and between insular subregions may contribute to the cognitive, emotional symptoms, and sensory information observed in individuals with SCD (Zang et al., 2007; Li et al., 2017). Additionally, insular dysfunction has been implicated in the pathophysiology of Alzheimer’s disease and other neurodegenerative disorders (Zhang and Li, 2012). Despite these findings, a comprehensive investigation into the altered functional connectivity of insular subregions in SCD is lacking, particularly regarding specific connectivity patterns within the anterior and posterior insula. The relationship between changes in functional connectivity and clinical cognition remains to be understood.

Therefore, the aim of the present study is to elucidate the altered functional connectivity patterns within the insular subregions in individuals with SCD using resting-state functional magnetic resonance imaging (rs-fMRI).

## 2 Materials and methods

### 2.1 Subjects

This study was conducted in accordance with the guidelines established by the Medical Research Ethics Committee of Aerospace Center Hospital. All participants provided written informed consent in line with the Declaration of Helsinki. The protocol was approved by the Medical Research Ethics Committee of Aerospace Center Hospital.

In total, 58 right-handed subjects participated in the study, comprising 30 patients with SCD and 28 healthy controls (HCs). The SCD subjects were randomly recruited from patients seeking treatment at the memory clinic at Aerospace Center Hospital for memory complaints. SCD was defined using the following question: “Over the past 12 months, have you experienced confusion or memory loss that is occurring more frequently or worsening?” This question was exclusively posed to participants aged 45 and above. It has been used in numerous studies (Brown et al., 2023) as a measure of SCD within the previous year. The SCD variable was categorized as “Yes” vs. “No.” The HCs were recruited from the local community through recruitment advertisements. All participants completed a standardized form, which included demographic information such as age, gender, and education, along with clinical history, family genetic history, previous examination results, and other pertinent clinical information.

The HCs met the following criteria: (a) no abnormal findings on routine brain MRI; (b) no history of stroke, depression, or epilepsy, and no other neurological or psychiatric disorders; (c) no visual loss, hearing loss, or other neurological deficits; (d) no reported cognitive or memory complaints; and (e) a Clinical Dementia Rating (CDR) score of 0. Participants with contraindications for MRI were excluded based on exclusion criteria.

All participants underwent a comprehensive physical examination, neurological assessment, and neuropsychological evaluation. The neuropsychological assessments included the Mini-Mental State Examination (MMSE), Activities of Daily Living Scale (ADL), World Health Organization-University of California-Los Angeles Auditory Verbal Learning Test (AVLT), Clinical Dementia Rating (CDR) score, Montreal Cognitive Assessment (MOCA), Clock Drawing Task (CDT), Hachinski Ischemic Score (HIS), and other tests. The clinical data for the remaining 58 participants are presented in Table 1.

**Table 1:** Demographic and neuropsychological test.

Variable	Total (*n* = 58)	Group		Statistic	*p*-value
		HC (*n* = 28)	SCD (*n* = 30)		
Age, mean ± SD	63.26 ± 4.75	64.39 ± 4.76	62.20 ± 4.57	*t* = 1.790	0.079
Education, mean ± SD	13.47 ± 4.02	14.00 ± 3.25	12.97 ± 4.62	*t* = 0.978	0.332
Gender, *n* (%)				*χ*^2^ = 0.000	0.986
Female	31 (53.45)	15 (53.57)	16 (53.33)		
Male	27 (46.55)	13 (46.43)	14 (46.67)		
MMSE 30, mean ± SD	28.52 ± 1.82	29.93 ± 0.26	27.20 ± 1.65	*t* = 8.946	<0.001
MOCA 30, mean ± SD	27.78 ± 2.82	29.75 ± 0.80	25.93 ± 2.78	*t* = 7.211	<0.001
AVLT, mean ± SD	55.09 ± 9.99	62.00 ± 7.20	48.63 ± 7.66	*t* = 6.836	<0.001
HIS 18, mean ± SD	1.34 ± 1.75	0.61 ± 0.79	2.03 ± 2.11	*t* = −3.456	0.001
CDT, mean ± SD	3.69 ± 0.78	3.93 ± 0.26	3.47 ± 1.01	*t* = 2.423	0.021
GDS 30, mean ± SD	1.60 ± 2.25	0.50 ± 0.84	2.63 ± 2.65	*t* = −4.197	<0.001
ADL 23, mean ± SD	20.74 ± 0.64	21.00 ± 0.00	20.50 ± 0.82	*t* = 3.340	0.002
HAMA 7, mean ± SD	1.53 ± 2.43	0.71 ± 1.88	2.30 ± 2.65	*t* = −2.638	0.011
CDR 0, mean ± SD	0.04 ± 0.14	0.00 ± 0.00	0.08 ± 0.19	*t* = −2.408	0.023

SCD, subjective cognitive decline; MMSE, Mini-Mental State Examination; MOCA, Montreal Cognitive Assessment; AVLT, World Health Organization-University of California-Los Angeles Aauditory Verbal Learning Test; HIS, Hachinski Ischemic Score; CDT, clock drawing task; GDS, Geriatric Depression Scale; ADL, Activity of Daily Living Scale; HAMA, Hamilton Anxious Scale; CDR, Clinical Dementia Rating; HC, healthy controls.

### 2.2 Data acquisition

The MRI examinations were conducted at the department of radiology using a 3.0T Siemens Prisma MR System (Siemens, Germany) with a 64-channel head coil. Foam padding and headphones were utilized to restrict head movement and minimize scanner noise. The rs-fMRI data were acquired axially using an echo-planar imaging (EPI) sequence: Repetition time (TR)/echo time (TE)/flip angle (FA) = 2,000 ms/30 ms/90°, field of view = 224 mm × 224 mm, image matrix = 112 × 112, slices = 62, thickness = 2 mm, gap = 0.3 mm, voxel size = 2 mm × 2 mm × 2 mm, and bandwidth = 2,230 Hz/pixel. The configuration data were collected using a 3D sagittal T1-weighted magnetization-prepared rapid gradient echo (MPRAGE) sequence: Time of Repetition (TR)/Time of Echo (TE)/Time of Inversion (TI)/Flip Angle (FA) = 2,530 ms/2.98 ms/1,100 ms/7°, slice number = 192, thickness = 1 mm, voxel size = 1 mm × 1 mm × 1 mm, field of view = 256 mm × 256 mm.

### 2.3 Data preprocessing

The resting-state functional magnetic resonance imaging (rs-fMRI) data preprocessing was conducted using the Statistical Parametric Mapping (SPM12) and Data Processing Assistant for rs-fMRI (Chao-Gan and Yu-Feng, 2010) toolkits. Data Preprocessing: fMRI data preprocessing was performed using standard procedures. Specifically, the first 10 volumes were removed, slice timing correction and head motion correction were applied. Next, the T1-weighted images were coregistered with the functional images and then registered into Montreal Neurological Institute (MNI) space. The normalized functional images were created by transforming the T1 images to a customized T1 template, with the functional images then resampled to 3 mm isotropic voxels. Spatial smoothing was applied using a 4 mm full width-half maximum Gaussian kernel. To reduce low-frequency drifts and high-frequency physiological noise, linear detrending and temporal bandpass filtering (0.01–0.1 Hz) were conducted. Nuisance variables, including six head motion parameters, cerebrospinal fluid, and white matter, were removed using multiple linear regression analysis. During image preprocessing, two healthy controls were excluded due to head motion exceeding defined thresholds (translation greater than 2.5 mm or rotation greater than 2.5°).

### 2.4 Definition of insular subregions

**Figure 1:**
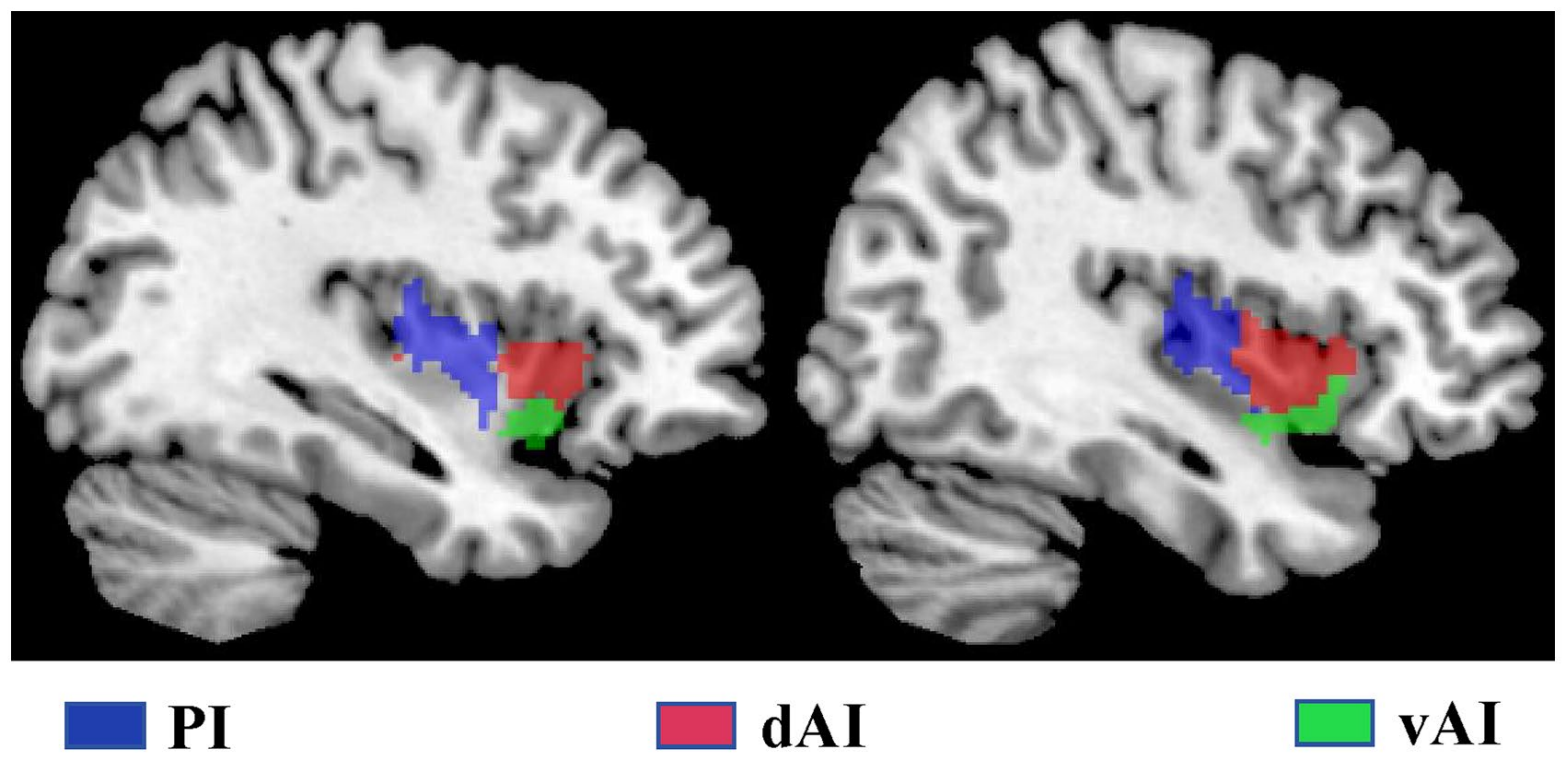
The subregions of the insula include the left and right insula, comprising the dorsal anterior insula (dAI) in red, the ventral anterior insula (vAI) in green, and the posterior insula (PI) in purple.

We characterized three insular subregions in each hemisphere according to a previous connection-based parcellation study (Deen et al., 2011). Figure 1 illustrates the three insular subregions in each hemisphere: ventral anterior insula (vAI), dorsal anterior insula (dAI), and posterior insula (PI).

### 2.5 Functional connectivity analysis of the insular subregions

For each subject, we calculated the Pearson’s correlation coefficients between the mean time series of each voxel within the insular subregion and the time series of all voxels in the other gray matter regions. These correlation coefficients were then converted to *z*-values using Fisher’s *r*-to-*z* transformation to improve normality. Finally, six *z*-score maps representing the intrinsic RSFC patterns of the six insular subregions were generated for each subject.

### 2.6 Statistical analysis

For each group, individuals’ *z*-values were entered into a random-effects one-sample *t*-test in a voxel-wise manner to detect brain regions that exhibited significant positive or negative correlations with each insular subregion. These analyses were corrected for false discovery rate (FDR) to account for multiple comparisons (*p* < 0.05, two-tailed). To compare intergroup differences in insular RSFC patterns after controlling for age, gender, and education, two-sample *t*-tests were conducted, respectively. The statistical significance threshold was set at *p* < 0.05 with correction using the Gaussian random field (GRF) method. To examine the relationship between the RSFC of each insular subregion with significant intergroup differences and cognitive behaviors, Spearman’s correlation coefficients were calculated between these RSFC values and cognitive behavior scores, while controlling for age, gender, and education as covariates. The statistical significance level was set at *p* < 0.05.

## 3 Results

### 3.1 Demographic and neuropsychological characteristics

Demographic and neuropsychological characteristics are detailed in Table 1. There were no significant differences in gender, educational level, and age between the SCD and HCs groups (all *p* > 0.05). However, significant differences were observed in the MMSE, AVLT, MOCA, CDT, GDS, ADL, CDR, HAMA, and HIS scores between the SCD and HCs groups (all *p* < 0.05).

### 3.2 Within-group RSFCs of the insular subregions

**Figure 2:**
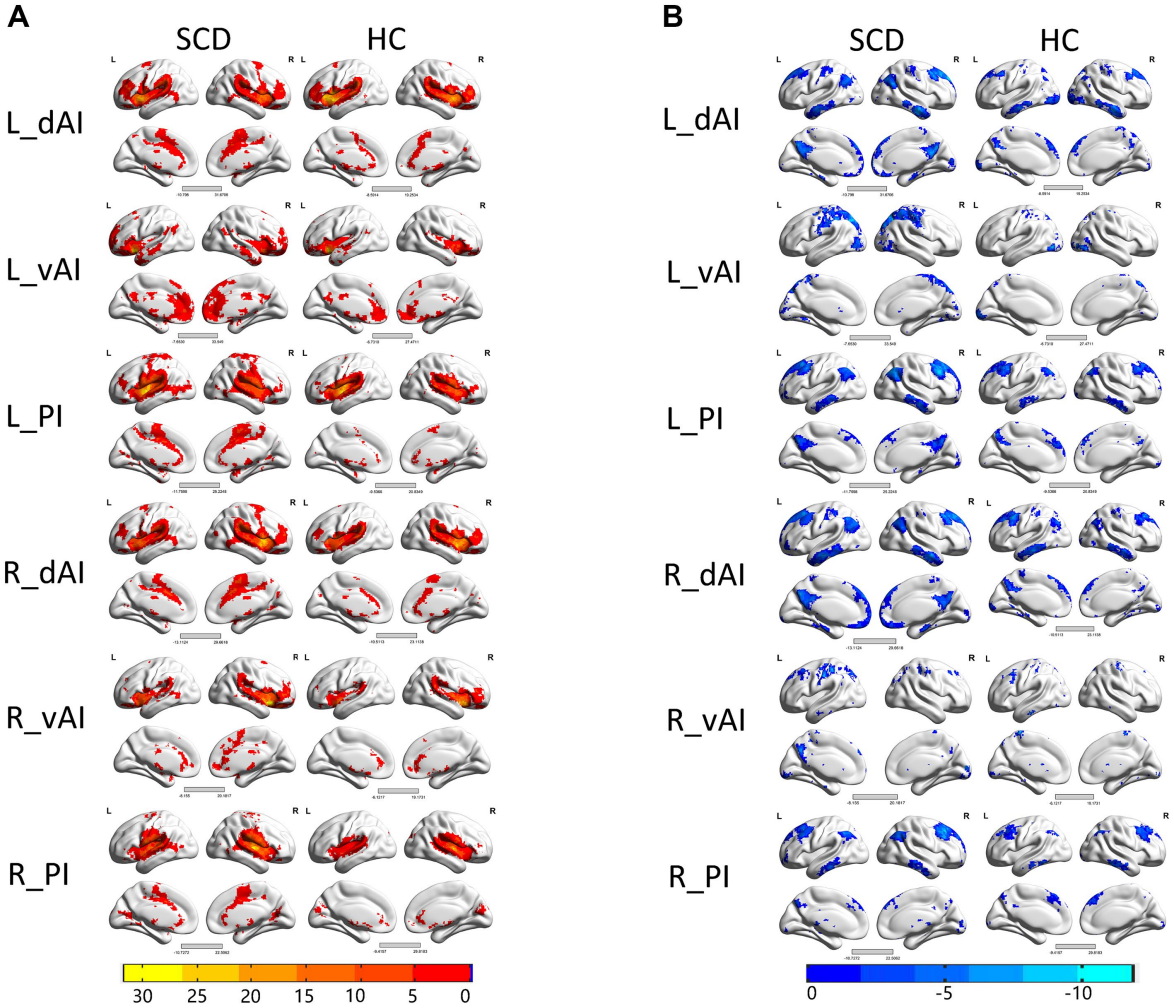
Within the group resting-state functional connectivity (RSFC) analysis, we identified **(A)** positive RSFC patterns of the insular subregions in each group, including healthy controls (HCs) and patients with subjective cognitive decline (SCD) (*p* < 0.05, false discovery rate (FDR) corrected); and **(B)** negative RSFC patterns of the insular subregions in each group, including HCs and patients with subjective cognitive decline (SCD) (*p* < 0.05, FDR corrected).

Figure 2 illustrates the functional connectivity maps for each insular subregion within the HC and SCD groups. Upon visual inspection, the connectivity maps within the HC and SCD groups exhibited similar patterns. Among the three subregions of the left insula, their positive connections are distinct. Specifically, in the left insula of the HC group, the dAI showed positive connectivity with the ECN regions, including the insula, superior frontal gyrus (SFG), supramarginal gyrus, middle temporal gyrus (MTG), inferior frontal gyrus (IFG), and middle cingulate cortex (MCC). Additionally, negative connectivity was observed in the default mode network (DMN) regions, such as the precuneus, medial prefrontal cortex (MPFC), angular gyrus (AG), and inferior temporal gyrus (ITG). The vAI displayed positive connectivity with the SN regions, which included the anterior cingulate cortex (ACC), insular cortex, medial orbitofrontal gyrus, superior temporal gyrus (STG), MTG, and putamen. Negative connectivity was predominantly observed in regions such as the precuneus, superior occipital gyrus (SOG), cerebellum, and pericalcarine cortex, many of which are part of the DMN. The posterior zone of the insula (PI) is typically considered a component of the SMN. Positive connectivity was observed with regions such as the insula, postcentral gyrus, precentral gyrus, STG, MTG, and supplementary motor area. Notably, the PI demonstrated significant negative connectivity with the precuneus, angular gyrus, cerebellum, MPFC, posterior cingulate cortex (PCC), and middle occipital gyrus (MOG), forming the classical pattern of the DMN.

### 3.3 Between-group RSFC differences of the insular subregions

**Figure 3:**
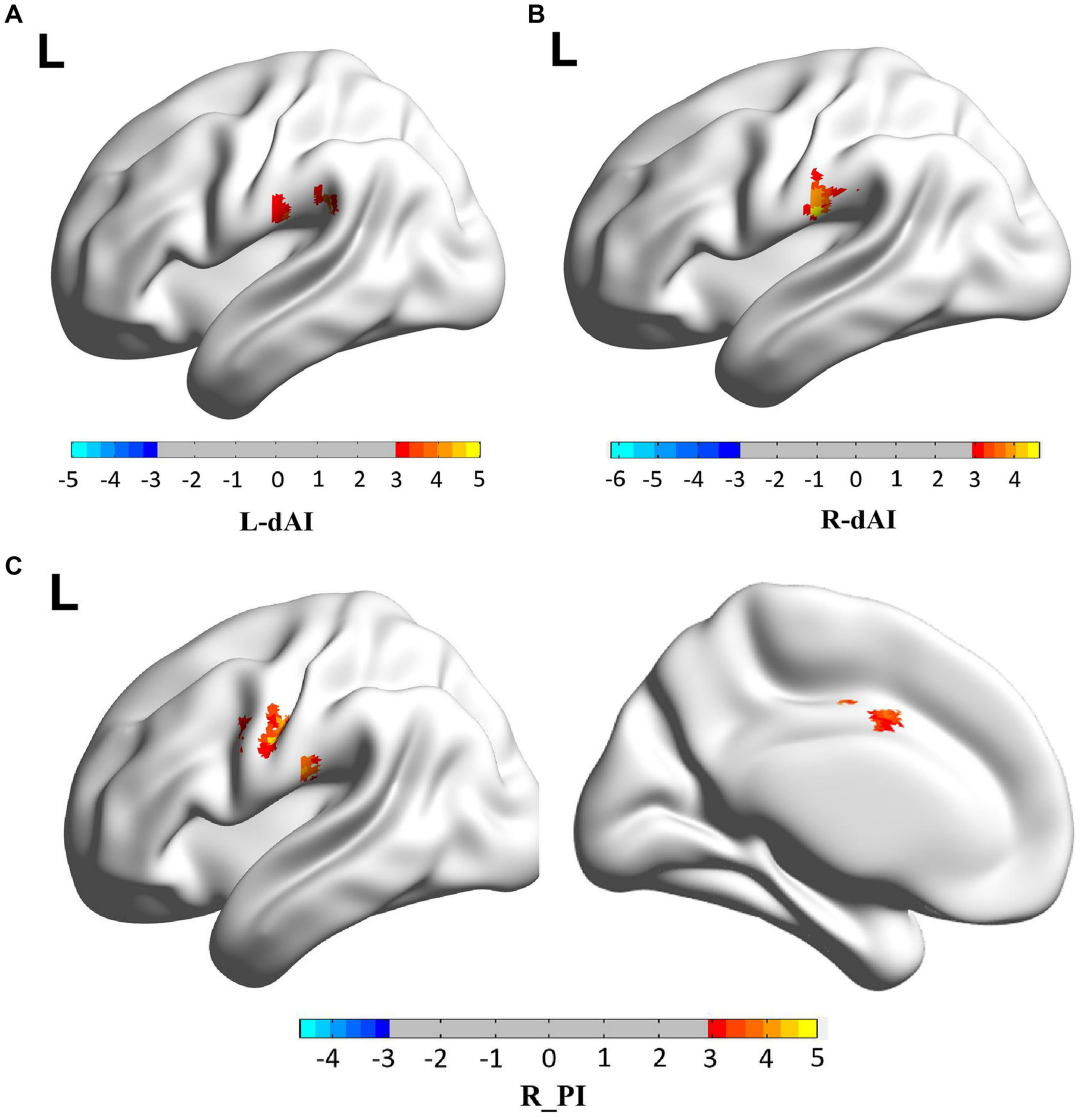
The enhanced positive RSFCs of insular subregions were observed in patients with SCD (*p* < 0.05, corrected using the Gaussian random field method). Different brain regions between SCD group and HC group are mainly concentrated between the ECN and SMN networks. **(A)** FC of left dAI subregion between SCD and HC patients, with the difference mainly concentrated in SupraMarginal_L. **(B)** FC of left vAI subregion between SCD and HC patients, with the difference mainly concentrated in Postcentral_L. **(C)** FC of right PI subregion between SCD and HC patients, the differences were mainly concentrated in Postcentral_L, Precentral_L, Cingulum_Mid_L.

When the two-sample *t*-test was performed with L. dAI as the seed region, the SCD group and the HC group showed increased positives in SupraMarginal_L, and this brain regions belonged to the ECN region. When the two-sample *t*-test was performed with R. dAI as the seed region, the SCD group showed increased positive in Postcentral_L with the HC group, which belonged to the SMN region. When the two-sample *t*-test was performed with R. PI as the seed region, the SCD group and the HC group showed increased positives in Postcentral_L, Precentral_L, and Cingulum_Mid_L, and most of these three brain regions belonged to the SMN region. In conclusion, the differences between the two groups are mainly concentrated between the ECN and SMN networks. And I also found that when the two-sample *t*-test was performed with L. dAI as the seed region, the SCD group showed increased negative in Cerebelum_Crus1 with the HC group. However, when the two-sample *t*-test was performed with R. vAI, L. vAI, L. PI as the seed area, no difference was found between the SCD group and the HC group. Figure 3 and Table 2 illustrate the between-group differences in positive functional connectivity for each insula subregion.

**Table 2:** Regions showing increased SCD-related RSFCs in insular subregions.

ROIs	Brain regions	Cluster size (voxels)	MNI Coordinates (mm)			Maximum *Z*
			*X*	*Y*	*Z*	
**L. dAI**						
Cluster 1	SupraMarginal_L	37	−57	−36	27	4.43
Cluster 3	Cerebellum_Crus1_R	26	42	−48	−36	4.48
**R. dAI**						
Cluster 1	Postcentral_L	35	−60	−18	21	4.52
**R. PI**						
Cluster 1	Postcentral_L	20	−66	−12	27	4.50
Cluster 2	Precentral_L	30	−53	−7	31	4.91
Cluster 3	Cingulum_Mid_L	26	−9	6	30	4.40

### 3.4 Relationship between the insular subregional RSFCs and cognitive performances

**Figure 4:**
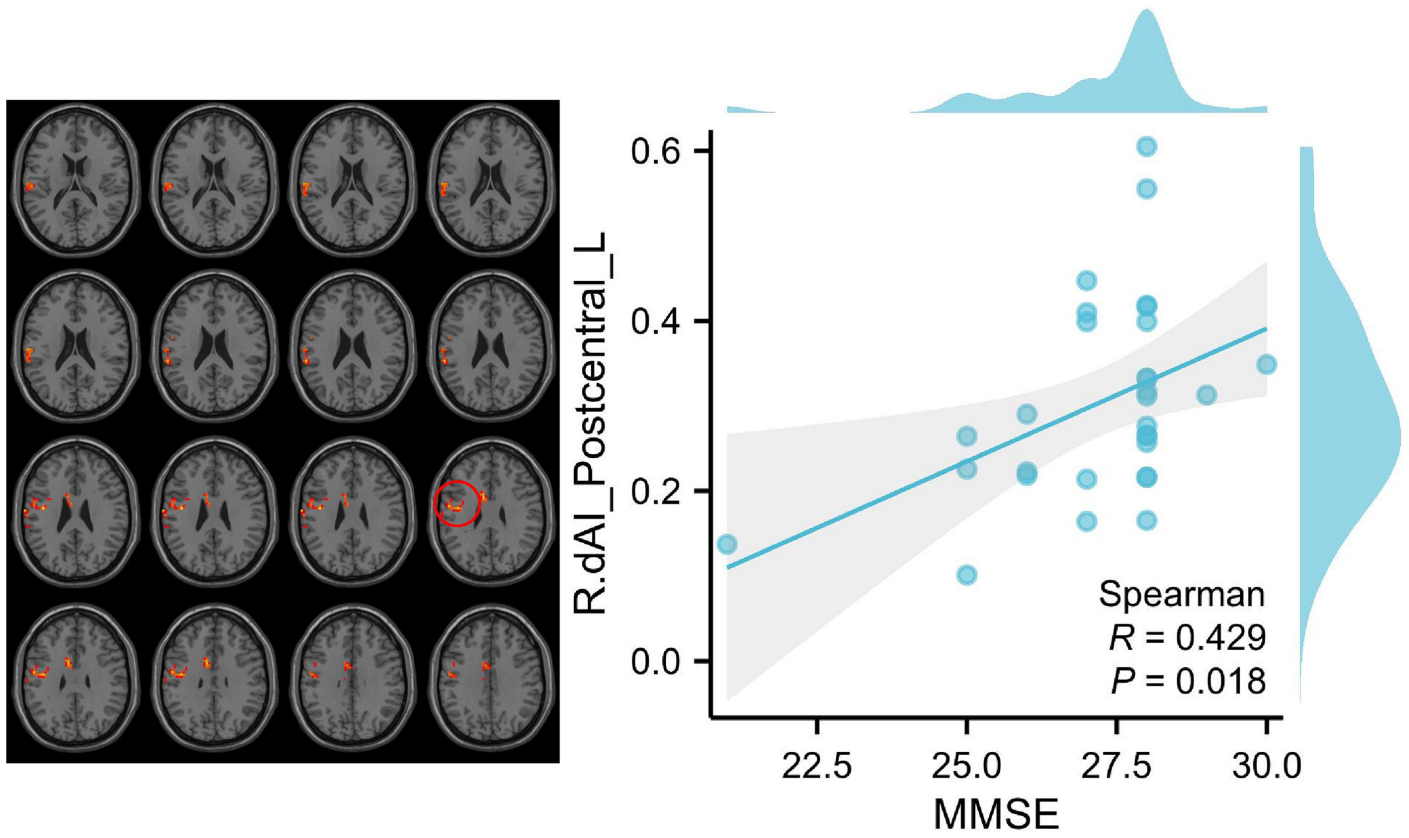
Correlation between clinical variables and RSFCs of insular subregions in patients with SCD. We analyzed the correlation between nine questionnaires, including MMSE, MoCA, AVLT, HIS, CDT, GDS, ADL, HAMA, and CDR, with differential brain regions in the SCD group and HC group, respectively. However, only MMSE was positively correlated with the functional connection between Postcentral_L and R_dAI (*p* = 0.018 < 0.05). The red circle indicates the Postcentral_L.

In order to empirically validate the behavioral significance of altered functional connectivity (FC) within insular subnetworks in patients with SCD, a correlation analysis was conducted to determine the relationship between abnormal FC patterns of insula subregions, cognition, and clinical measures, while controlling for age, sex, and education. We analyzed the correlation between nine questionnaires, including MMSE, MoCA, AVLT, HIS, CDT, GDS, ADL, HAMA, and CDR, with differential brain regions in the SCD group and HC group, respectively. However, only MMSE was positively correlated with the functional connection between Postcentral_L and R_dAI (*p* = 0.018 < 0.05) (Figure 4).

## 4 Discussion

Functional connectivity refers to the synchronization of neural activity between brain regions, reflecting the strength of communication and coordination between them. In the current study, we analyzed resting-state fMRI data acquired from both HCs participants and individuals SCD to demonstrate distinctive patterns of RSFC in each subregion of the insula. We identified three cognitive-related RSFC patterns involving positive connectivity within the insula, suggesting anterior-to-posterior functional subdivisions. Specifically, we observed that the dAI exhibited positive connectivity with regions of the ECN, including the insula, superior frontal gyrus (SFG), supramarginal gyrus, middle temporal gyrus (MTG), inferior frontal gyrus (IFG), and middle cingulate cortex (MCC). The vAI was positively associated with regions of the SN, including the anterior cingulate cortex (ACC), insular cortex, medial orbitofrontal gyrus, superior temporal gyrus (STG), middle temporal gyrus (MTG), and putamen. The PI was traditionally considered a component of the SMN, with positive connectivity shown with the insula, postcentral gyrus, precentral gyrus, STG, MTG, and supplementary motor area. Comparison between patients with SCD and HCs revealed the following differences. SCD patients exhibited increased in positive RSFC in different networks (executive control network and SMN). There were no differences in positive RSFC within the SN between the two groups. Furthermore, we observed that changes in insular connectivity were to a certain extent correlated with cognitive performance in SCD patients.

The investigation into the altered functional connectivity of insular subregions in SCD represents a significant advancement in our understanding of the early neural changes associated with cognitive impairment. The insular cortex, a region involved in cognitive, emotional, and interoceptive functions, has been implicated in SCD. The study revealed that these insular subregions exhibit connectivity patterns associated with the ECN, SN, and SMN, which are implicated in various cognitive and affective functions.

### 4.1 Insular cortex and subregions

The insular cortex, also known as the insula, is a complex brain region located in the lateral sulcus with connections to various cortical and subcortical areas. It is traditionally divided into anterior and posterior subregions, each serving distinct functions. The anterior insula is involved in emotional processing, social cognition, and cognitive control, while the posterior insula plays a role in somatosensory processing and interoception. The insula acts as an integrative hub, processing internal and external stimuli to regulate behavior and emotional responses. Understanding the functional connectivity within and between insular subregions is essential for unraveling the neural mechanisms underlying cognitive decline in SCD.

### 4.2 Distinct connectivity patterns in insular subregions

The study identified distinct connectivity patterns in various sub-regions of the insula, shedding light on the functional segregation and integration within this complex brain region. Specifically, the anterior dorsal insula, known for its role in emotional processing and cognitive control, showed connectivity with the ECN, which is responsible for goal-directed behavior and working memory. In our previous studies, the ECN network within the insula included the MTG, DLPFC, IFG, IPL, ACC, and MCC. We found similarities with previous studies in defining the ECN network, but there were differences in areas such as the SupraMarginal region. We attribute these variations to differences in sample sizes and demographics.

In contrast, the SN, which mediates the detection of behaviorally relevant stimuli and regulates attention, exhibited altered connectivity with the anterior ventral insula in individuals with SCD. Several previous studies have identified the SN in humans, showing consistent activation during both internally and externally emotional stimuli (Damoiseaux et al., 2006; Seeley et al., 2007; Uddin et al., 2011). This network consists of paralimbic structures, most notably the ACC and orbital frontal insula, extending to the basal ganglia and striatum, responsible for guiding thought and behavior (Seeley et al., 2007; Uddin et al., 2010). Our study revealed positive connectivity within the group between the vAI and the ACC, insular, Frontal_Mid_Orb, STG, Frontal_Med_Orb, MTG, and putamen, which overlapped with the SN regions. In the PI connectivity analysis, we identified associated regions such as the insula, postcentral, precentral, and Supp_Motor_Area, suggesting the involvement of the SMN. This network had connectivity with primary sensory and motor cortices, indicating the importance of related function (Beckmann et al., 2005; De Luca et al., 2006; van den Heuvel et al., 2008). Although our study focused on the positive correlations, we also observed that in the within-group insula connectivity analysis, most regions negatively connected to the insula overlapped with regions of the DMN. Decreased connectivity between the anterior insula and regions of the DMN indicates disruptions in self-referential processing and cognitive control.

### 4.3 Altered functional connectivity in SCD

No functional connectivity reduction area was found. Increased positive RSFCs in SCD patients were found in several specific regions associated with the ECN and SMN, while alterations in the SN regions were not observed in SCD patients relative to HCs. Since SCD is an early stage disease, these changes may suggest early compensatory adaptations. These alterations may underlie abnormalities in salience attribution and attentional processes in cognitive decline. Additionally, changes in connectivity between the posterior insula and sensory regions may reflect disturbances in interoceptive awareness and sensory processing. In previous studies, decreased activation of the supplementary motor area and premotor cortex has been reported in AD patients using motor-related tasks (Agosta et al., 2010; Vidoni et al., 2012). However, in our SCD patient population, the SMN region did not exhibit decreased connectivity as seen in AD patients, but rather showed enhancement, suggesting that decreased SMN connectivity in AD patients may be a sign of decompensation. Our study found enhanced functional connectivity between the left anterior insular dorsalis and the left cerebellum, contrary to the findings of Wang et al. (2021). Additionally, early studies have shown that the cerebellar posterior lobe contributes to episodic memory encoding (Fliessbach et al., 2007). We hypothesize that due to the early stage of SCD in patients, there may be a certain degree of compensation in the cerebellar region to enhance memory encoding. Moreover, Wang et al. (2021) identified compensatory brain regions in SCD patients, including the middle frontal gyrus (MFG), middle temporal gyrus (MTG), and inferior frontal gyrus (IFG). However, in our study, the regions of compensation were different, primarily in the SMN and ECN regions, including the SupraMarginal_L, Cerebellum_Crus1_R, Postcentral_L, Precentral_L, and Postcentral_L regions. Due to the limited number of relevant studies, further research is needed to confirm these findings in the future.

Furthermore, the SMN, responsible for motor planning and execution, exhibited connectivity changes with specific insular subregions, suggesting potential motor-related deficits in SCD patients. The varying involvement of these functional brain networks in insular connectivity underscores the diversity of cognitive impairments in SCD and emphasizes the requirement for network-specific interventions customized to individual symptom profiles. These findings underscore the potential impact of insular connectivity alterations on the pathophysiology of SCD.

### 4.4 Cerebellum and cognitive impairment

It used to be conventional wisdom cognitive decline occurs due to structural or functional abnormalities in the cerebral cortex, there are few studies on the relationship between cerebellum and cognitive impairment. The cerebellum, traditionally known for its role in motor coordination, is now recognized as playing a crucial role in cognitive functions as well. In our study, we found that when the two-sample *t*-test was performed with R. dAI as the seed region, the SCD group showed increased negative in Cerebellum_Crus1 with the HC group. This enhancement may be a compensatory alteration in the early stages of the disease, which also provides a new reference for the compensatory regions of early brain functional connectivity in SCD patients. Research has shown that the cerebellum is involved in aspects of cognition such as language, attention, working memory, and executive functions (Stoodley and Schmahmann, 2009). While the exact mechanisms by which the cerebellum contributes to these cognitive processes are still being elucidated, evidence suggests that disruptions in cerebellar function can lead to deficits in cognitive performance. Studies have found associations between cerebellar lesions and deficits in cognitive function. For example, a study by Schmahmann and Sherman (1997) reported that patients with cerebellar lesions exhibited impairments in executive functions such as planning, set-shifting, and abstract reasoning. Furthermore, a meta-analysis by Stoodley and Schmahmann (2009) found evidence that individuals with cerebellar dysfunction showed deficits in attention, working memory, and language processing. Moreover, neuroimaging studies have provided insights into the neural networks involved in cerebellar-cognitive interactions. Functional MRI studies have shown that during cognitive tasks, the cerebellum is activated in conjunction with cortical regions such as the prefrontal cortex and parietal cortex (Strick et al., 2009). Connectivity analyses have further demonstrated the existence of cerebro-cerebellar loops that are involved in information processing and integration during cognitive tasks (Stoodley et al., 2012).

### 4.5 Correlation between RSFCs and cognitive performances

Our results demonstrate a correlation between RSFC of the dAI and clusters in the postcentral gyrus with Mini-Mental State Examination (MMSE) scores in patients with SCD. Specifically, SCD patients with higher MMSE scores exhibit increased RSFC between the right dAI and clusters in the postcentral gyrus. This finding is in line with previous studies on the ECN and SMN, both of which show compensatory mechanisms maintaining normal clinical function by enhancing functional connectivity.

### 4.6 Implications for understanding SCD pathophysiology

The increased of ECN connectivity with the insula may lead to transform in cognitive control, decision-making, and attentional processes, compensatory to memory lapses and executive dysfunction in SCD. Moreover, alterations in SMN connectivity with specific insular subregions may manifest as motor coordination difficulties or sensorimotor impairments in individuals with SCD. Understanding these network-specific connectivity patterns provides a neurobiological basis for the diverse cognitive and behavioral symptoms observed in SCD and informs targeted interventions to address these deficits.

### 4.7 Future directions and challenges

While the study’s findings provide valuable insights into the connectivity patterns of insular subregions in SCD, several future research directions and challenges warrant attention. Longitudinal studies tracking changes in insular connectivity over time in individuals with SCD are essential to elucidate the progression of network-specific alterations and their relationship to cognitive decline. Investigating the interactions between insular connectivity patterns and other brain regions or networks implicated in SCD, such as the DMN or frontoparietal network (FPN), could provide a more comprehensive understanding of the neural mechanisms underlying cognitive impairment. Integrating multi-modal imaging techniques, such as functional magnetic resonance imaging (fMRI), diffusion tensor imaging (DTI), and electroencephalography (EEG), may offer a more nuanced assessment of insular connectivity and its functional relevance in SCD. Furthermore, validating the clinical utility of network-specific insular connectivity biomarkers and interventions through large-scale clinical trials is crucial for translating research findings into effective diagnostic and therapeutic strategies for individuals with SCD. Collaboration among researchers, clinicians, and industry partners will be instrumental in overcoming these challenges and advancing the field of network neuroscience in cognitive decline.

### 4.8 Clinical relevance and practical applications

The analysis of altered functional connectivity of insular subregions in SCD has important clinical relevance and practical applications. Neuroimaging biomarkers derived from insular connectivity patterns may assist clinicians in early detection and monitoring of cognitive decline in at-risk individuals. These biomarkers could provide additional insights into the underlying neural changes associated with SCD and complement traditional clinical assessments. Interventions targeting insular connectivity networks could serve as innovative approaches to delay or prevent cognitive decline in individuals with SCD. By modulating insular connectivity through targeted interventions, clinicians may enhance cognitive function and quality of life in individuals experiencing subjective cognitive complaints.

### 4.9 Limitations

There are several limitations that need to be considered in our study. First, in the current study, we only focused on resting state fMRI data. In the future, we can combine multimodal MRI methods such as fMRI, DTI, and perfusion to analyze the brain function, structure, and blood flow of SCD patients simultaneously, which may be helpful for a deeper understanding of the mechanisms of SCD. Second, small sample size. Third, we did not gather physiological measurements, gene data, or other biological recordings due to experimental design and equipment restrictions. In the future, we will collect gene data, cerebral spinal fluid (CSF), as well as images of amyloid-β plaques to acquire more comprehensive experimental data.

## 5 Conclusion

In conclusion, this study has demonstrated that different sub-regions of the insula can exhibit varying connectivity patterns, associated with distinct functional brain networks such as the ECN, SN, and SMN, which are impacted differently in patients with SCD. The analysis of altered functional connectivity of insular subregions in SCD provides insight into the early neural changes linked to cognitive decline. Through the exploration of connectivity patterns within and between insular subregions, researchers have the potential to develop new diagnostic tools, biomarkers, and interventions for individuals at risk of neurodegenerative diseases. Further research in this area offers promise for enhancing early detection, personalized interventions, and collaborative efforts to preserve cognitive function and improve quality of life in aging populations experiencing cognitive decline. The investigation of insular connectivity alterations in SCD underscores the significance of early intervention strategies and interdisciplinary approaches in addressing cognitive impairment.

## Data availability statement

The original contributions presented in the study are included in the article/supplementary material, further inquiries can be directed to the corresponding author.

## Ethics statement

The studies involving humans were approved by Aerospace Center Hospital, Beijing, China. The studies were conducted in accordance with the local legislation and institutional requirements. The participants provided their written informed consent to participate in this study.

## Author contributions

HT: Conceptualization, Data curation, Investigation, Methodology, Software, Visualization, Writing – original draft. WZ: Methodology, Supervision, Validation, Writing – review & editing. JW: Methodology, Supervision, Validation, Writing – review & editing. SL: Methodology, Supervision, Validation, Writing – review & editing. ZW: Supervision, Validation, Writing – review & editing.
